# Sustainable volume sweep imaging lung teleultrasound in Peru: Public health perspectives from a new frontier in expanding access to imaging

**DOI:** 10.3389/frhs.2023.1002208

**Published:** 2023-04-03

**Authors:** Thomas J. Marini, Benjamin Castaneda, Malavika Satheesh, Yu T. Zhao, C. Mahony Reátegui-Rivera, Walter Sifuentes, Timothy M. Baran, Katherine A. Kaproth-Joslin, Robert Ambrosini, Gloria Rios-Mayhua, Ann M. Dozier

**Affiliations:** ^1^ Department of Imaging Sciences, University of Rochester Medical Center, Rochester, NY, United States; ^2^ Departamento de Ingeniería, Laboratorio de Imágenes Médicas, Pontificia Universidad Católica del Perú, Lima, Peru; ^3^ Medical Innovation & Technology, Lima, Perú; ^4^ Department of Public Health, University of Rochester Medical Center, Rochester, NY, United States

**Keywords:** global health, lung ultrasound, pneumonia, pulmonary disease, telemedicine, ultrasound

## Abstract

**Background:**

Pulmonary disease is a common cause of morbidity and mortality, but the majority of the people in the world lack access to diagnostic imaging for its assessment. We conducted an implementation assessment of a potentially sustainable and cost-effective model for delivery of volume sweep imaging (VSI) lung teleultrasound in Peru. This model allows image acquisition by individuals without prior ultrasound experience after only a few hours of training.

**Methods:**

Lung teleultrasound was implemented at 5 sites in rural Peru after a few hours of installation and staff training. Patients were offered free lung VSI teleultrasound examination for concerns of respiratory illness or research purposes. After ultrasound examination, patients were surveyed regarding their experience. Health staff and members of the implementation team also participated in separate interviews detailing their views of the teleultrasound system which were systematically analyzed for key themes.

**Results:**

Patients and staff rated their experience with lung teleultrasound as overwhelmingly positive. The lung teleultrasound system was viewed as a potential way to improve access to imaging and the health of rural communities. Detailed interviews with the implementation team revealed obstacles to implementation important for consideration such as gaps in lung ultrasound understanding.

**Conclusions:**

Lung VSI teleultrasound was successfully deployed to 5 health centers in rural Peru. Implementation assessment revealed enthusiasm for the system among members of the community along with important areas of consideration for future teleultrasound deployment. This system offers a potential means to increase access to imaging for pulmonary illness and improve the health of the global community.

## Introduction

Respiratory disease, acute and chronic, is a major cause of morbidity and mortality around the world (1–3). In children under 5 years of age, pneumonia remains the leading cause of mortality (4, 5). Diagnostic imaging is critical to diagnosing many respiratory illnesses including pneumonia, as symptoms of respiratory disease such as fever and cough are non-specific (6, 7). However, the majority of the world lacks access to diagnostic imaging (8–11). To bridge this gap, low-cost lung ultrasound imaging could be employed. Lung ultrasound is highly sensitive and specific for the detection of many respiratory diseases including pneumonia, pleural effusion, and pulmonary edema (12–20).

Even with low-cost hand-held ultrasound, deployment of lung ultrasound is limited by the availability of trained staff to perform and interpret examinations particularly in low- and middle-income countries. Teleultrasound offers one means to overcome these issues but remains constrained by availability of specialists, system ease of use, and limited healthcare infrastructure among other obstacles (21, 22). To overcome obstacles to deploy teleultrasound and increase its use, a new user-friendly teleultrasound system that requires neither high-speed internet nor an ultrasound specialist has been successfully piloted utilizing volume sweep imaging (VSI) (23). This approach has already demonstrated excellent performance in controlled clinical trials for obstetric, right upper quadrant, and thyroid scanning indications (23–26). Testing of lung and breast VSI has also shown excellent clinical efficacy (27–29).

**Figure 1:**
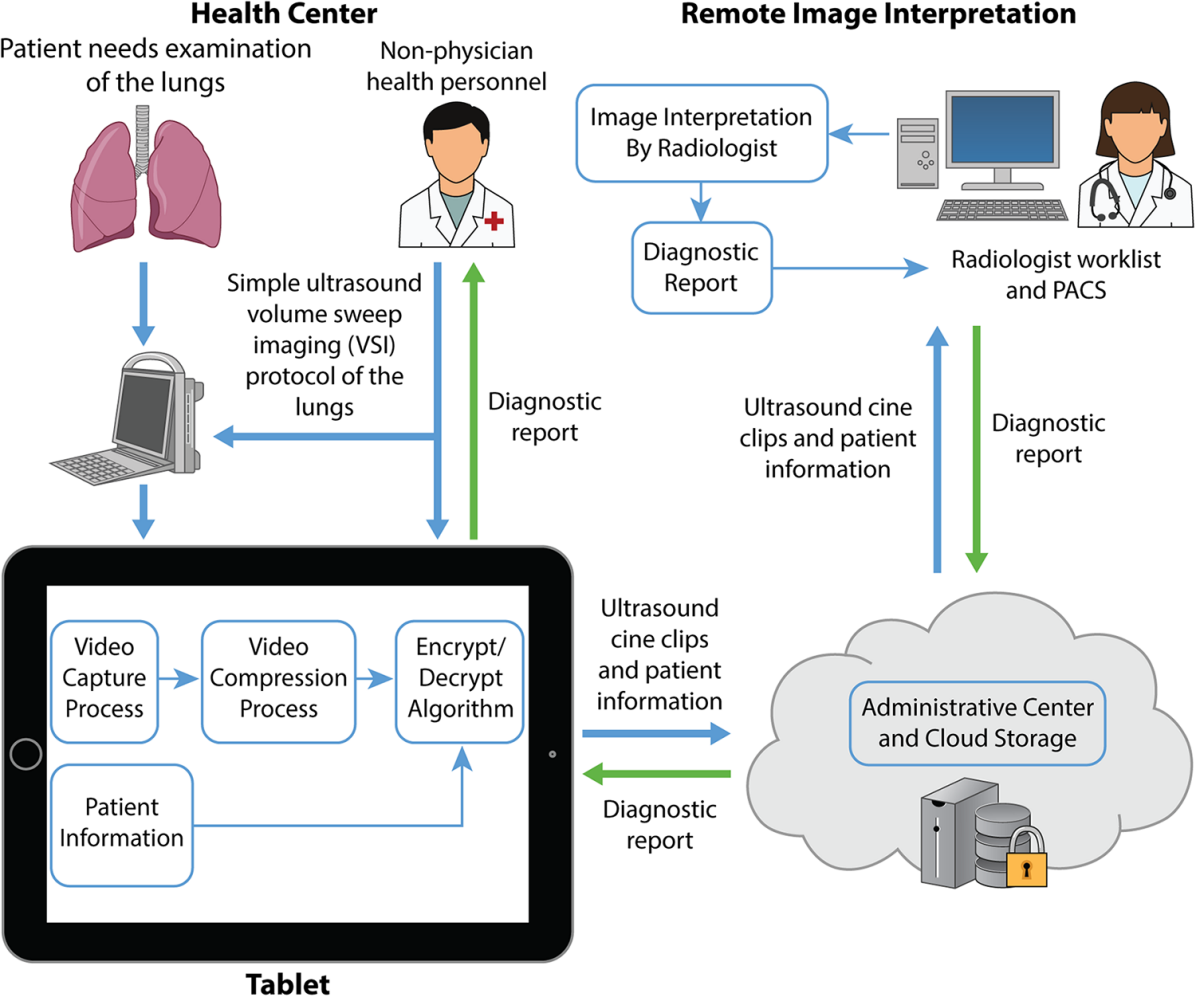
Asynchronous VSI lung teleultrasound. Schematic diagramming the components of the lung teleultrasound system utilized in this study. In practice, patients presenting to clinic with an indication for lung imaging would receive a lung VSI exam performed by an individual at the health center. The operator performing the scan may be an individual without prior ultrasound or medical experience. The tablet guides the user to perform the VSI protocol and input relevant patient history which is uploaded to a secure cloud for download by a specialist remotely. The specialist uses the history and images to produce a diagnostic report which is sent back to the health center to be shared with the clinic team.

Building on this success, lung VSI teleultrasound was deployed at several sites in rural Peru during the COVID-19 pandemic (Figure 1) (29). While there is established clinical efficacy of lung ultrasound VSI and clearly great theoretical clinical benefit to the use of lung VSI teleultrasound in practice, there are many questions that remain to be elucidated in regards to integration of lung VSI teleultrasound into clinical practice. We undertook an implementation assessment to inform how such a teleultrasound program could be integrated into the larger healthcare milieu. Specifically, we aimed to analyze the benefits, drawbacks, considerations, and challenges for implementation of this lung teleultrasound system. We speculated that there would be perceived excellent benefits to implementation complicated by logistical challenges such as lack of education regarding lung ultrasound. The goal of this work was to inspire further public health investigation into the potential of this approach to improve global health.

## Deployment of teleultrasound in rural Peru

### Teleultrasound system

The teleultrasound system used in study activities has been previously described in detail, and a diagram demonstrating its application for lung ultrasound is shown in Figure 1 (23). Briefly, a user-friendly telemedicine application is installed on a tablet which connects to an ultrasound machine. This application guides the user to enter patient information as well as perform each step of the VSI ultrasound protocol. The tablet screen captures the ultrasound machine screen and saves the data from each sweep of the ultrasound probe over the target anatomy. This system is completely asynchronous meaning it can acquire images in the absence of a radiologist or specialist. Images can also be acquired in environments without internet and stored locally until an internet connection is available. The report from the radiologist is sent back to the tablet to be shared with the patient and health workers.

Imaging is acquired with the lung VSI ultrasound protocol (Supplementary Figure S1). VSI is an imaging technique in which an individual with minimal prior ultrasound training performs a specialized scan protocol based on external body landmarks requiring neither significant technical skill or anatomical knowledge (30, 31). The imaging protocol consists of a series of blind sweeps of the ultrasound probe over the thorax. The video of each sweep is saved for expert interpretation and sent via the telemedicine platform. The operator performing the scan does not interpret the imaging. Lung VSI has also been previously shown to be easily taught at a rural Peruvian health center over the course of a few hours (31). A clinical trial of lung ultrasound VSI previously showed 100% sensitivity and 93% specificity for pneumonia (27).

### Teleultrasound deployment

The activities conducted in this study were approved by the institutional review board at the Hospital Cayetano Heredia in Peru and began in November 2020. Along with opportunity to evaluate pulmonary pathology, the COVID-19 pandemic posed logistical study challenges that were overcome as effectively as possible. The study was funded by the mining company Nexa Resources as a service to the communities they operate within. The 5 sites for the study were chosen by the mining company based on their sites of corporate operation, not the prevalence of pneumonia or scientific considerations (Supplementary Figure S2).

The communities where the teleultrasound system was deployed were in the Peruvian departments of Ancash, Ica, and Pasco. The communities in Ancash were located in Conchucos and Pampas. The community in Ica was Chavín. The communities in Pasco were San Juan de Milpo and Ticlacayán. Elevation in these areas is greater than 3,000 m. According to the Peruvian Ministry of Health, population in each of these areas is on the scale of a few thousand people with the number using each health center even smaller. Imaging was not readily available to these communities prior to the installation of the teleultrasound program. To obtain an imaging exam, transportation would need to be acquired to the closest metropolitan center.

**Figure 2:**
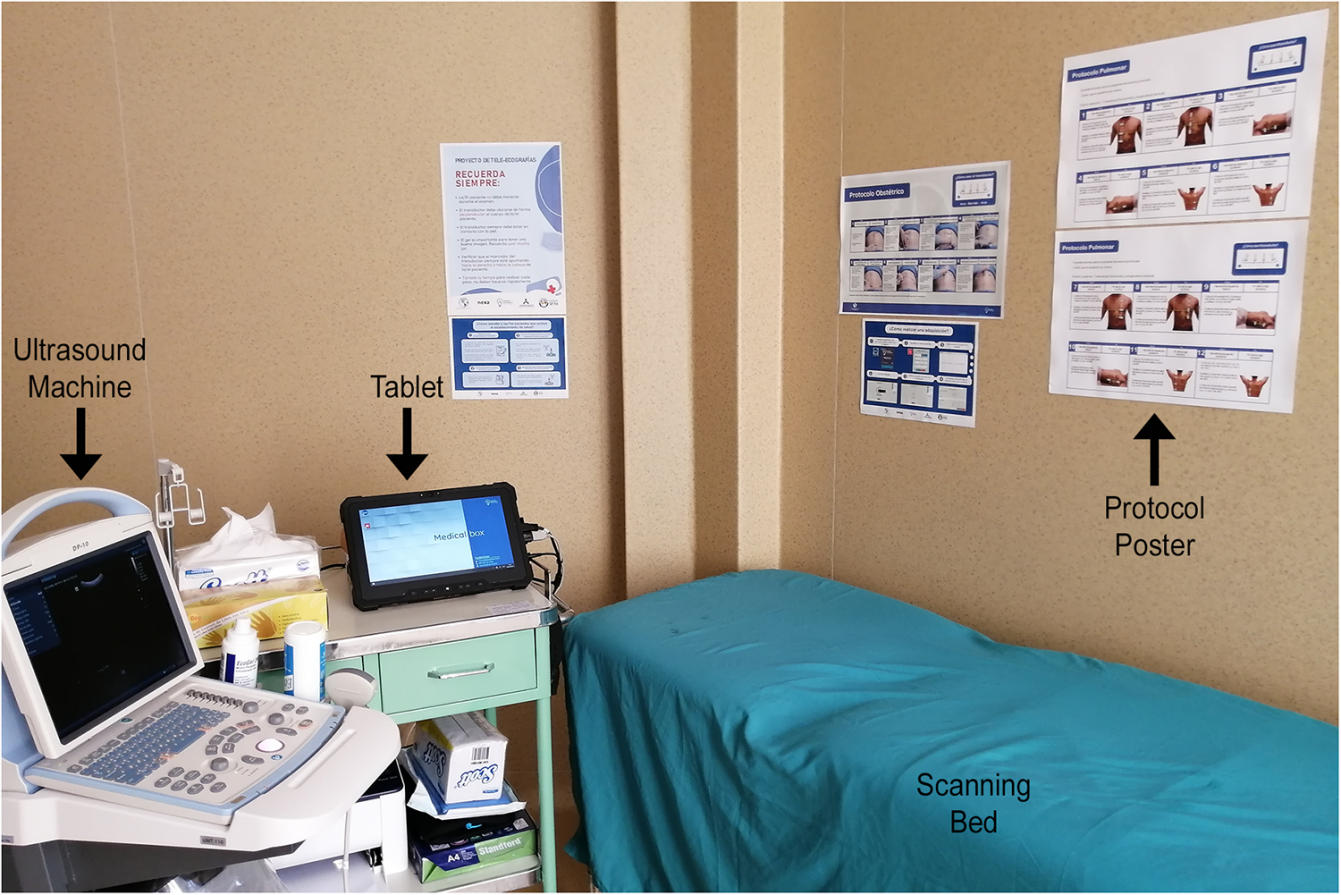
Example teleultrasound station. Labeled photo from the teleultrasound station at Ticlacayán demonstrating the required setup. The tablet connects to the ultrasound machine and guides the user to input the clinical history and perform the VSI protocol. Posters on the wall remind the operator how to perform the protocol.

At all sites, the telemedicine system was set up at a small health post offering basic health services by a team from the Peruvian company Medical Innovation and Technology. The set-up of the teleultrasound unit used at the Ticlacayán site is shown in Figure 2. A photograph of the exterior at Ticlacayán is shown in Supplementary Material S1. In Pasco, the teleultrasound services were also advertised by radio. Trainers traveled to each of the 5 sites and installed the telemedicine system and educated staff. Staff training typically occurred in the span of approximately 8 h over the course of 3 days and involved didactic and hands-on training sessions. Individuals have previously demonstrated basic competence with the lung VSI protocol over the course of a few hours (31). At the end of training, every operator was certified to perform the protocol correctly. Those trained included clinic technicians and nurses.

Patients attending each site were offered a free lung VSI teleultrasound exam for general research purposes or concern of respiratory illness. Upon enrollment, the operator performed the VSI protocol described above, entering the patient’s clinical history into the tablet and obtaining the images. The images were transmitted for radiologist reading and the results returned to the health center (to be shared with the patient and healthcare provider). During the initial phase of the study for analysis in this paper, 213 patients were scanned with the lung teleultrasound system (147 female and 66 male). Their average age was 42.8 years (standard deviation 18.2 years, range 0–92 years). The average turnaround time for results was 18.8 h (standard deviation 29.3 h, range: 2–279 h). Of those scanned, 43.2% (*n* = 92) were symptomatic and 56% (*n* = 121) were asymptomatic. Exams were performed on symptomatic patients for many reasons including cough, shortness of breath, and fever/chills.

The clinical results of these examinations have been previously published and thoroughly described elsewhere (29). Cardiothoracic radiologists rated 202 out of the 213 examinations as diagnostic in image quality with only a single truly non-diagnostic exam secondary to a technical error. Among a random subset of these exams, the radiologists had 91% agreement on lung ultrasound interpretation with all discrepancies in agreement related to borderline examinations straddling the line between normal and abnormal. Clinical analysis showed the lung teleultrasound system was able to diagnose sequalae of COVID-19 infection. There were *n* = 15 patients with abnormal ultrasound examinations and *n* = 29 patients with borderline examinations straddling the continuum between normal and abnormal requiring clinical correlation and appropriate follow-up.

## Implementation assessment

Concurrent to the study clinical activities, an assessment of the implementation was undertaken. Data were systematically collected from enrolled patients through a structured patient survey administered by the clinic staff in the local language. The survey included open-ended items about patient characteristics, such as the reason for coming to the clinic, whether they were aware of the new service, how they heard about it, and their experience with it.

In addition, clinic staff completed a brief survey after their training on the protocol. The survey queried their perceptions about the teleultrasound system and its potential impact using open-ended questions. Their experience with the training was assessed using 4 items and analyzed using univariate statistics. Responses to the open-ended questions were aggregated and common themes identified.

In addition, more detailed interviews, (conducted via video) were completed with a staff member in Pasco and from those involved with implementation and training, teleultrasound system development, and reading the submitted images. Interviews were conducted in a free-format with open-ended questions regarding the teleultrasound system and implementation. Questions were tailored to the role of the person being interviewed. These interviews were analyzed similarly to the above open-ended questions.

## Results

Using the patient survey, 67 patients who had participated in the lung ultrasound scan were interviewed. The results are summarized in Supplementary Table S1. Slightly more than half (55%; *n* = 37) were aware of the teleultrasound service before arrival to the health center. Reasons for coming to the center included obtaining teleultrasound (48%; *n* = 32) and “COVID” (22%; *n* = 15). The majority of those who knew about the service learned about it from health personnel. At one site that used radio promotions, a few patients noted that they heard it about it via the radio. Patients uniformly rated their experience as good or very good (100%).

Among the 7 clinic staff who completed a survey, they universally viewed the teleultrasound as important and beneficial to the community/clinic. Example quotations include: “…it is important because we have limited accessibility to this service that we need in our community.”; “…it is going to be very helpful and very useful for... all rural areas.” Respondents also mentioned reducing the need for referral to a higher level of care, more timely diagnosis, and for those who provide pharmacotherapy, initiation of antibiotic treatment as possible benefits. Training was viewed positively with the majority giving the highest rating to each of the 4 questions (Supplementary Table S2). Their recommendations for promoting the availability of the service was to disseminate information through multiple means including social media (including radio), word of mouth, and through staff at the health center.

The more in-depth interviews included key members of Medical Innovation and Technology including their CEO, project administrator, and an employee with experience traveling to the health centers and training workers on lung VSI. Additionally, a Peruvian radiologist who read lung VSI scans as well as a member of the rural health post in Pasco were interviewed. Their comments fell into 4 key areas: program planning and site selection, staff training, advertising and outreach, and program implementation.

### Program planning

This process involved not only the health clinic but required pre-approval from the Regional Director of Health. Before training, signed contractual agreements had to be in place with the mining company; at some sites there were delays with changes in agreement wording and obtaining required signatures. Originally the process from site selection to implementation was expected to take 2–3 months. One person stated, “We were optimistic that we will go, and people will quickly sign and move and say they love it.” However, the reality was that the process took up to 6 months. The COVID-19 pandemic contributed to some of this delay as health centers had competing priorities, and planning in some cases had to shift to online as transportation to the selected remote sites was sometimes prohibited.

### Staff training

Interviews confirmed the above description of the few hours of training that includes demonstrations and practicing with the ultrasound device and the tablet. Depending on the site size, up to 5 individuals were trained. It typically took 3–4 attempts with the protocol to result in an error free set of images consistent with prior study (31). Doing the training online was viewed as inadequate (leading to errors in arcs and speed). Follow-up post-training was offered via video. Generally, if the first use of the teleultrasound is more than 2 weeks after the training an online refresher session was needed.

### Advertising and outreach

Within the health center, posters with simple to understand graphics were posted to advertise and explain the service (Supplementary Materials S2 and S3). While the local health center is closest (and less expensive), some community members prefer to go to the nearest urban area (which can be hours by car). Someone feeling very sick may skip the health center as the available services are limited and, in some cases, they would be referred elsewhere anyway. Having teleultrasound was thought to be a potential factor in helping to overcome this.

### Program implementation

While the potential benefits of this approach were met with general enthusiasm by clinic staff, some brought up concerns regarding implementation challenges including its integration into workflow. In general, there was an inverse relationship between staff viewing this service as extra work and its potential benefit to the community. One person stated, “[Some] health center staff looked at this as a compulsory activity of the health center. They looked at this as extra work, and they are not considering this as a help for the community.” Some staff asked for additional compensation for taking on this new responsibility.

The radiologist reading the scans had no direct contact with the sites but provides feedback about quality or other issues to the respective site. The radiologist reviews the images and generates a report which takes approximately 10 min per patient and is done remotely from a computer. This individual also needs to be specifically trained in the use of ultrasound for diagnosing lung disease which is not always a part of traditional radiology training.

## Discussion

Lung VSI teleultrasound performed by individuals without prior ultrasound training was feasible to implement in rural Peru. As respiratory disease remains a major cause of morbidity and mortality worldwide, this model for lung teleultrasound has a potentially vital role in expanding access to high-quality diagnostic imaging globally to underserved areas. The use of VSI and telemedicine circumvents issues relating to lack of specialists, prohibitive costs, and high-speed internet access. In this study, lung teleultrasound was successfully deployed within 8 h of training and produced turnaround times on average less than 24 h. These metrics suggest the approach is sustainable and scalable as a viable solution to improve access to imaging in remote sites. The primary clinical use of this system would be to detect or rule out respiratory disease including pneumonia, pleural effusion, and pulmonary edema. Lung ultrasound has been shown to be diagnostically superior to chest x-ray for many pulmonary conditions including pneumonia (21).

Our implementation assessment provided vital insight in regards to incorporating teleultrasound into remote clinics. There was general enthusiasm for VSI lung teleultrasound and universal acknowledgement of this approach as a means to improve health of local communities. Potential benefits of decreased delay to diagnosis and decreased transportation cost-savings to more urban clinics were frequently noted. Transportation to better equipped health centers can take up to days in Peru and is associated with worse health outcomes (32, 33). Staff training was accomplished through in-person didactic and hands-on sessions that were well-received and required limited follow-up beyond feedback after the first few post-training scans. Patients were motivated to come into the center to get a scan, and their perceptions were positive.

This study occurred during the COVID-19 pandemic. While this was an opportunity to test the system for detection of pulmonary disease, it also posed numerous logistical challenges. Despite these challenges, even in the midst of a global pandemic, the teleultrasound system was implemented successfully producing turnaround times of less than 24 h in rural Peru. Given the successful implementation in this context, lung VSI teleultrasound should be considered deployable in most situations.

Some interviewed reported patients with severe illness often “leapfrog” basic health centers to go to centers with more treatment capacity. This owes partly because in some of these locations, concern for pneumonia simply results in referral to a larger health center since the lower-resource health centers do not always have the capacity to treat pneumonia adequately. In this context, a negative teleultrasound could preempt a referral in sites which do not offer treatment, but from our interviews, it seems patients often would likely seek a higher level of care even if lung teleultrasound is available if they believe they are sick enough to require treatment. The incidence of respiratory disease, a health center’s capacity to treat pneumonia, distance to alternative treatment facilities, and local attitudes all should be considered when identifying sites for deployment. It is also possible that mobile teleultrasound units or screening campaigns could be a useful means to deploy teleultrasound to communities.

Another theme that emerged in our study was the vital role of government and public health policy in deployment. Partnership is needed with local governments and health centers to make lung teleultrasound financially feasible (Supplementary Material S4). While the cost of lung teleultrasound using our system is relatively low, teleultrasound will only be sustainable in the context that it is reimbursed. Reimbursements likely will need to come from government funding, but often the government may not reimburse for teleultrasound services adequately. In addition, health centers need to be adequately staffed and provided the appropriate resources to perform VSI teleultrasound. Some staff requested additional pay for taking on the responsibility of teleultrasound which is ultimately a public policy issue to address.

Lung VSI teleultrasound has the ability to improve the value of healthcare by increasing availability of ultrasound which is a relatively cheap imaging modality (34). This increased availability could lead to decreased morbidity and mortality by decreasing delays to diagnosis and allowing more accurate diagnosis further lowering costs of healthcare. To deliver lung VSI teleultrasound requires a tablet, brief training, and a portable ultrasound machine. Traditional ultrasound services also require an experienced operator to obtain images which is another dimension by which lung VSI teleultrasound can potentially decrease the cost of vital health services. In addition, as previously mentioned, there could be substantial cost savings to patients by decreasing the transportation costs associated with seeking care. Further dedicated economic analysis and modeling will be needed to fully assess the economic implications of VSI teleultrasound deployment.

In general, we found no insurmountable obstacles to implementation. Poor internet connectivity can limit efficiency of image transmission. However, as the teleultrasound system is asynchronous, images can be locally saved for transmission when a stable connection becomes available. Importantly, the teleultrasound system can send images at low internet bandwidths also mitigating the impact of poor connections (23). Although patients in this study were also scanned for general research purposes, we noted clinic staff sometimes seemed confused as to the appropriate indications for lung ultrasound scanning. Although these ultrasound scans are of low-cost and otherwise cause no harm to the patient, future efforts should be undertaken to develop educational material on lung ultrasound to increase general knowledge of the appropriate indications for lung ultrasound with clinic staff.

While this study demonstrated feasibility of implementation, there are additional knowledge gaps which remain in relation to implementation of lung teleultrasound. Future studies should examine the effect of such a program on health outcomes including morbidity and mortality. Economic analysis would again be helpful to identify potential cost-savings. There are also additional ancillary benefits to the deployment of lung ultrasound which may result from implementation such as increasing vaccination rates through increased clinic attendance. Ideally future studies will occur on a larger scale in areas with high rates of pathology as well as incorporate artificial intelligence (35, 36).

## Conclusion

This pilot of a potentially cost-effective and sustainable model for lung teleultrasound performed by individuals without prior ultrasound training demonstrated the feasibility of the approach and provided vital information regarding its implementation. A system offering diagnostic VSI lung teleultrasound scans and interpretations within 24 h was able to be implemented at 5 sites in rural Peru after minimal time investment and cost. Further public health study is needed to better delineate its impact on health outcomes in terms of morbidity and mortality, analyze economic benefit, and understand the best ways to integrate VSI teleultrasound into regular clinical practice. This user-friendly teleultrasound system’s ability to effectively image the lungs in the absence of a specialist or internet circumvents major obstacles that have limited the deployment of teleultrasound in the past. Its proper use could improve diagnosis and treatment of pulmonary disease to the benefit of global health.

## Data Availability

The original contributions presented in the study are included in the article/Supplemental Material. Further inquiries can be directed to the corresponding author.
