# Supplementary material for: Sustainable volume sweep imaging lung teleultrasound in Peru: Public health perspectives from a new frontier in expanding access to imaging
Source: Front Health Serv. 2023 Apr 3;3:1002208. doi: 10.3389/frhs.2023.1002208 (PMC10106710; doi:10.3389/frhs.2023.1002208)
Supplement: Supplementary file 1 [file Datasheet1.docx]

Supplemental Figures:


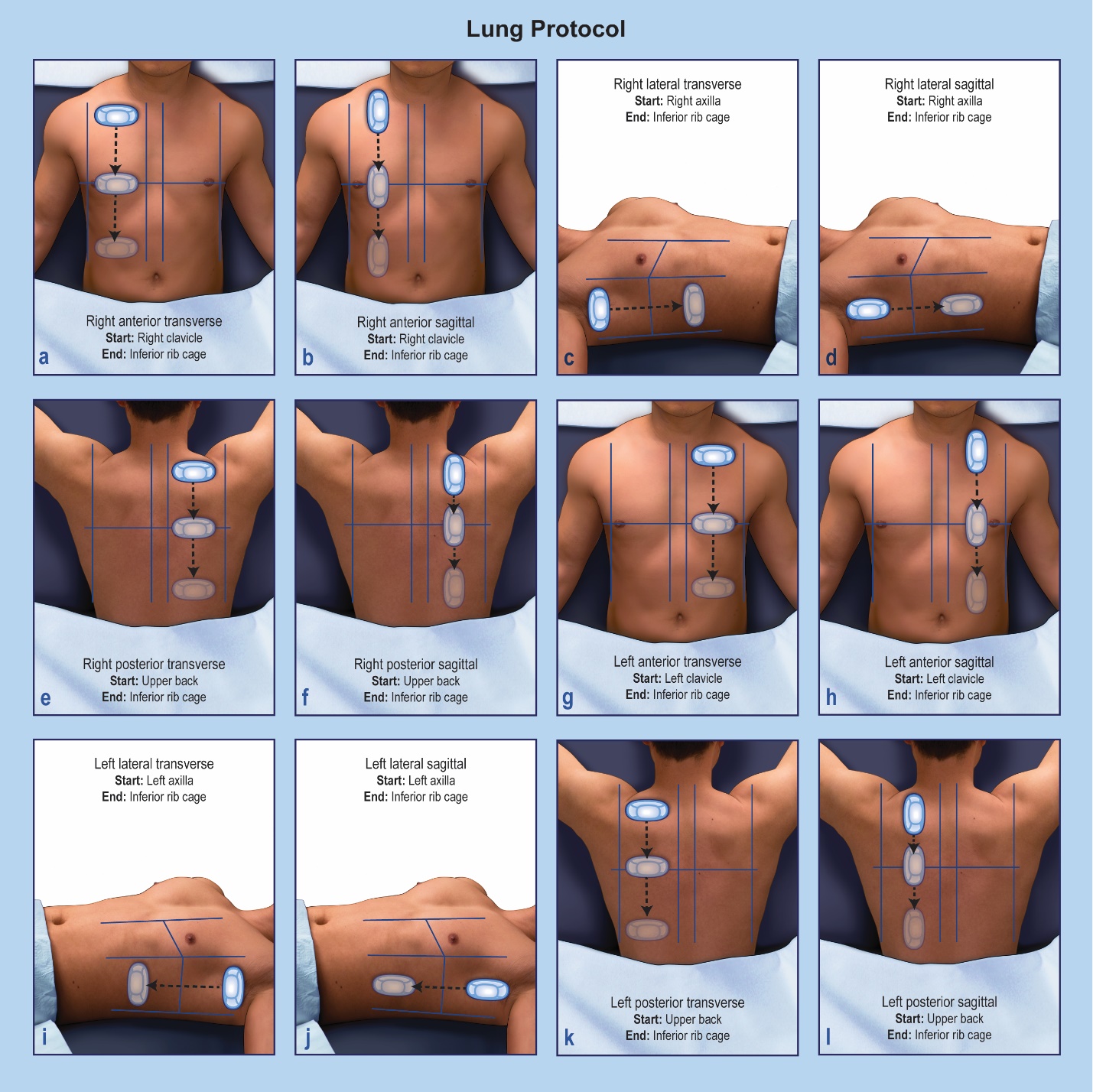


Supplemental Figure 1. Lung Volume Sweep Imaging Protocol. Schematic poster demonstrating how lung VSI is performed.


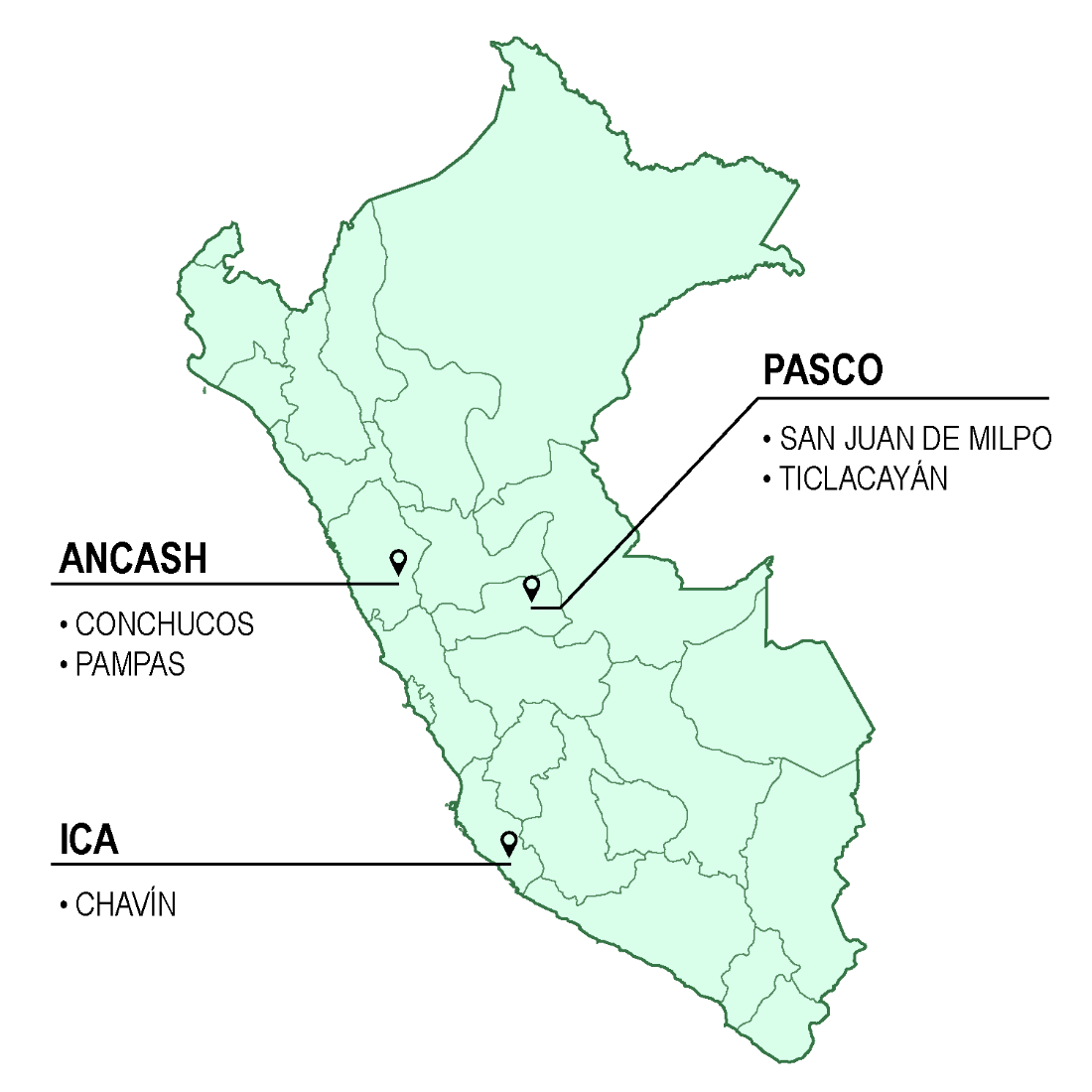


Supplemental Figure 2. Study Sites. Map of Peru showing sites of study activities.

Supplementary Material:


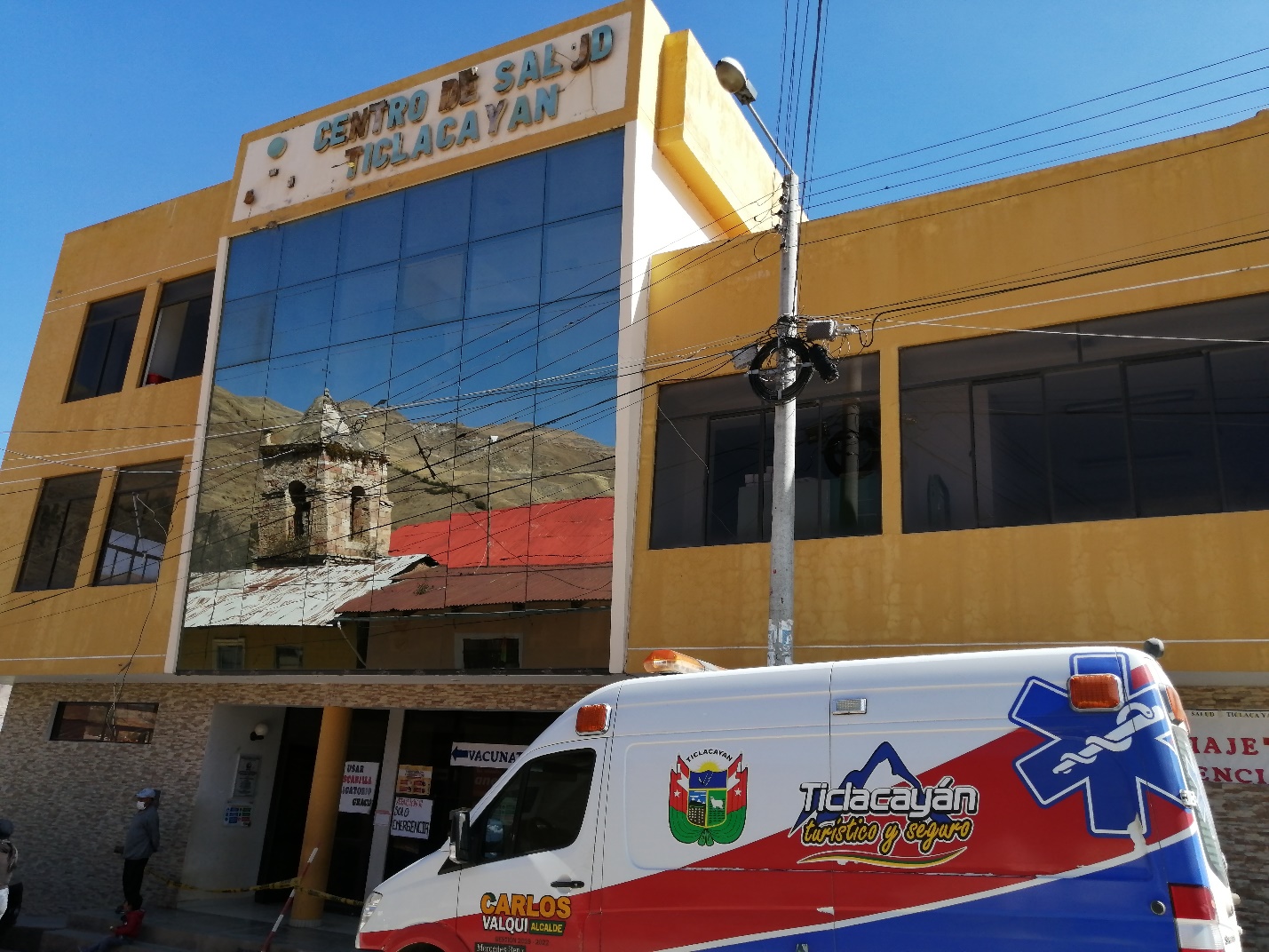


Supplemental Material 1. Photo of the Ticlacayán health center, one of the 5 study sites.


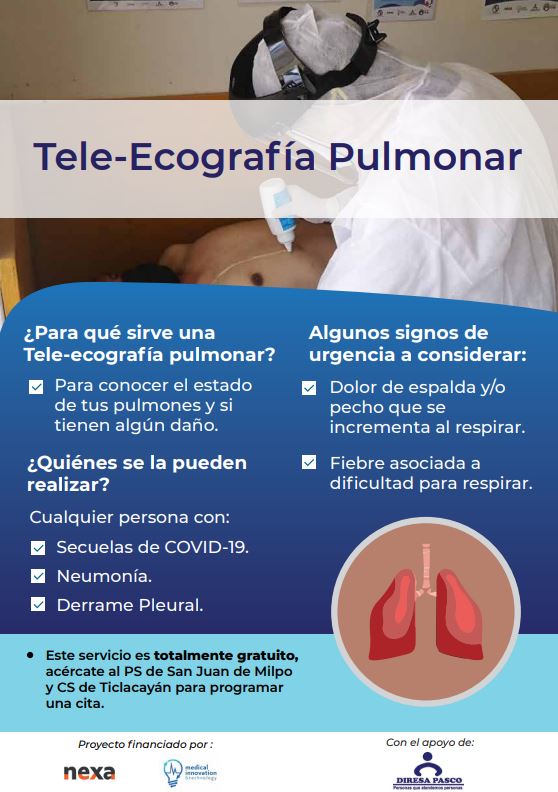


Supplemental Material 2. Study poster in Spanish advertising free lung ultrasound examinations and educating about the uses of lung ultrasound.


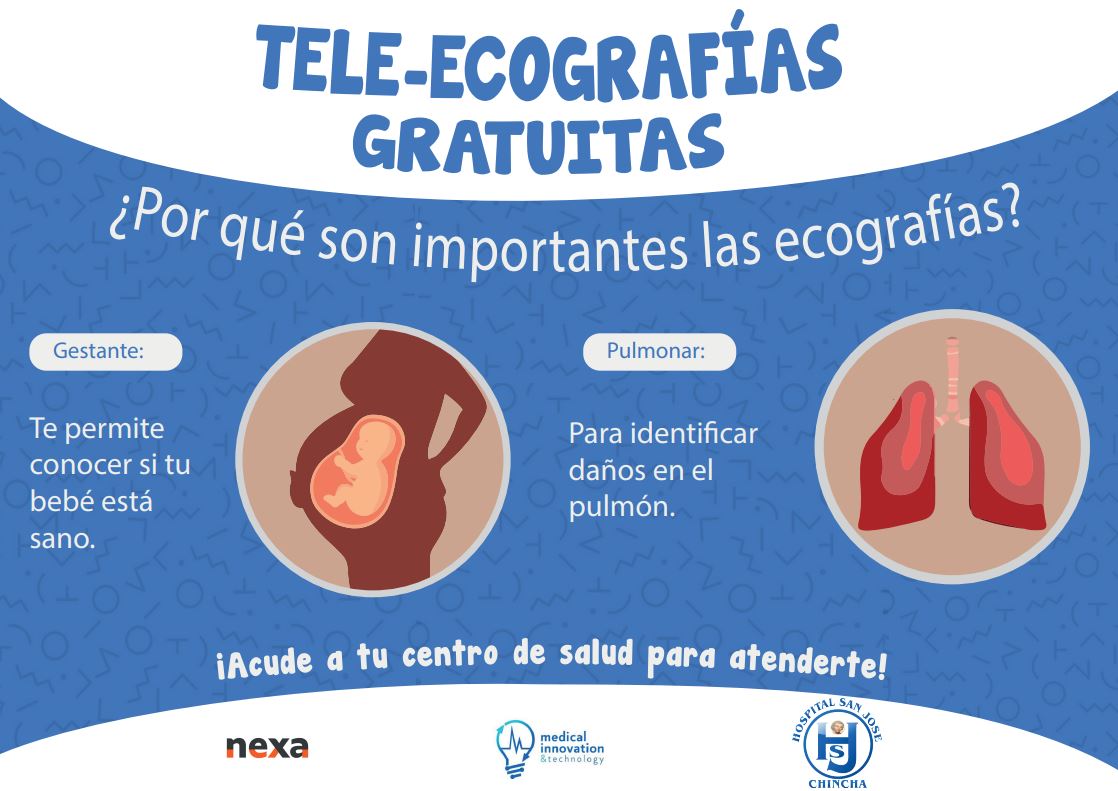


Supplemental Material 3. Study poster in Spanish advertising the use of lung and obstetric teleultrasound.


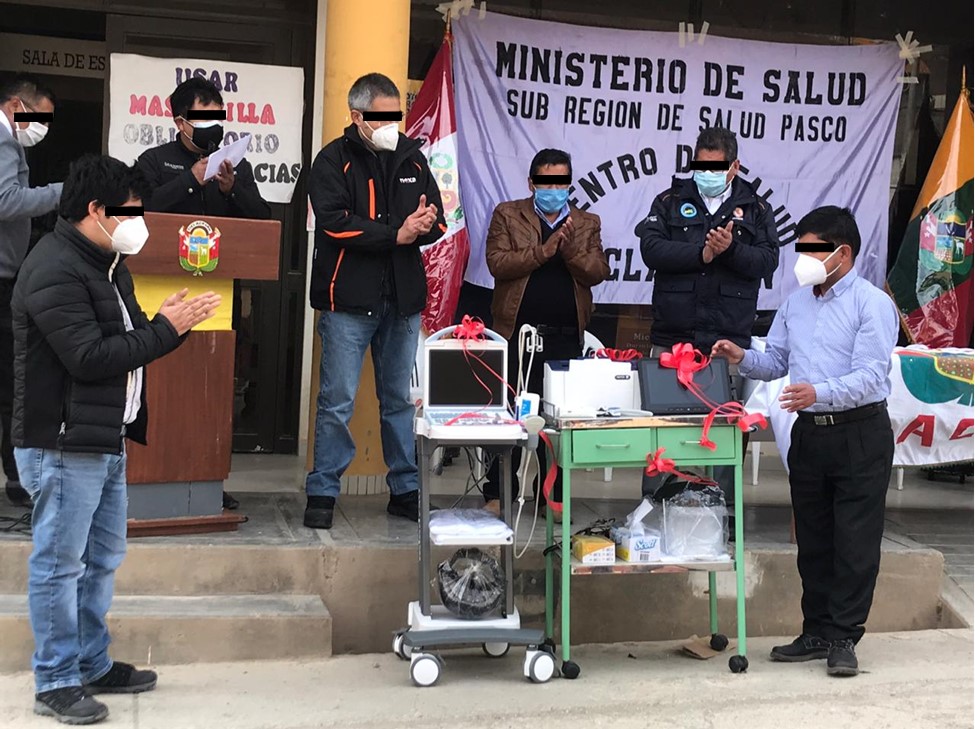


Supplemental Material 4. Photo from a ceremony celebrating the start of teleultrasound services at Ticlacayán. This occurred with partnership from the local health center and the regional government.
