# Supplementary material for: Sustainable volume sweep imaging lung teleultrasound in Peru: Public health perspectives from a new frontier in expanding access to imaging
Source: Front Health Serv. 2023 Apr 3;3:1002208. doi: 10.3389/frhs.2023.1002208 (PMC10106710; doi:10.3389/frhs.2023.1002208)
Supplement: Supplementary file 2 [file Datasheet2.docx]

Supplemental Table 1: Patient Survey Results (n=67). Patients rated the experience as “very bad,” “bad,” “good,” or “very good.”

| Variable | Category | Summary (n=67) * |
| --- | --- | --- |
| Age (years) | - | 39±17 (6-83) |
| Sex (%) | Not specified | 13% (6-24%, n=9/67) |
|  | Female | 55% (43-67%, n=37/67) |
|  | Male | 31% (21-44%, n=21/67) |
| Why did you come to the health center? | Not specified | 7% (2-17%, n=5/67) |
|  | Teleultrasound | 48% (35-60%, n=32/67) |
|  | COVID-19 | 22% (13-34%, n=15/67) |
|  | Back pain | 36% (25-49%, n=24/67) |
|  | Cough | 1% (0-8%, n=1/67) |
|  | Other | 4% (1-13%, n=3/67) |
| Did you know about the service? | Not specified | 9% (3-19%, n=6/67) |
|  | No | 36% (25-49%, n=24/67) |
|  | Yes | 55% (43-67%, n=37/67) |
| How did you find out about the service? | Not specified | 4% (1-13%, n=3/67) |
|  | Health center | 73% (61-83%, n=49/67) |
|  | Employment | 3% (0-10%, n=2/67) |
|  | From community member | 7% (2-17%, n=5/67) |
|  | From radio | 12% (5-22%, n=8/67) |
| How was the experience? | Not specified | 12% (5-22%, n=8/67) |
|  | Good | 10% (4-20%, n=7/67) |
|  | Very good | 78% (66-87%, n=52/67) |
| Center | Chavín | 3% (0-10%, n=2/67) |
|  | Milpo | 22% (13-34%, n=15/67) |
|  | Ticlacayán | 75% (63-85%, n=50/67) |

* Values are mean ± standard deviation (range) for continuous variables, and percentage (95% confidence interval, rate) for categorical variables. All values are rounded to the nearest whole number.

Supplemental Table 2. Staff Survey Results (n=7). Staff were able to answer “strongly disagree,” “disagree,” “neutral,” “agree,” or “strongly agree.”

| Variable | Category | Summary (n=7) * |
| --- | --- | --- |
| I trust my ability to obtain a VSI teleultrasound acquisition | Agree | 29% (4-71%, n=2/7) |
|  | Strongly Agree | 71% (29-96%, n=5/7) |
| I am satisfied with my training | Agree | 29% (4-71%, n=2/7) |
|  | Strongly Agree | 71% (29-96%, n=5/7) |
| Learning to obtain a teleultrasound is very easy | Agree | 14% (0-58%, n=1/7) |
|  | Strongly Agree | 86% (42-100%, n=6/7) |
| Learning to obtain a teleultrasound was something I enjoyed | Agree | 43% (10-82%, n=3/7) |
|  | Strongly Agree | 57% (18-90%, n=4/7) |

* Values are percentage (95% confidence interval, rate). All numbers are rounded to the nearest whole number.
